# Conformation and mechanical property of *rpoS* mRNA inhibitory stem studied by optical tweezers and X-ray scattering

**DOI:** 10.1371/journal.pone.0222938

**Published:** 2019-09-26

**Authors:** Xinyao Hu, Xuanling Li, Lingna Yang, Yilin Zhu, Yunyu Shi, Yinmei Li, Haowei Wang, Qingguo Gong

**Affiliations:** 1 Department of Optics and Optical Engineering, University of Science and Technology of China and Hefei National Laboratory for Physical Sciences at the Microscale, Hefei, Anhui, P. R. China; 2 School of Life Sciences, University of Science and Technology of China, Hefei, Anhui, P. R. China; Tsinghua University School of Life Sciences, CHINA

## Abstract

3′ downstream inhibitory stem plays a crucial role in locking *rpoS* mRNA 5' untranslated region in a self-inhibitory state. Here, we used optical tweezers to study the unfolding/refolding of *rpoS* inhibitory stem in the absence and presence of Mg^2+^. We found adding Mg^2+^ decreased the free energy of the RNA junction without re-arranging its secondary structure, through confirming that this RNA formed a canonical RNA three-way junction. We suspected increased free energy might change the relative orientation of different stems of *rpoS* and confirmed this by small angle X-ray scattering. Such changed conformation may improve Hfq-bridged annealing between sRNA and *rpoS* RNA inhibitory stem. We established a convenient route to analyze the changes of RNA conformation and folding dynamics by combining optical tweezers with X-ray scattering methods. This route can be easily applied in the studies of other RNA structure and ligand-RNA.

## Introduction

Bacteria developed a global stressors response to various environmental stress including high temperature, starvation, osmotic pressure, and unsuitable pH during life cycle [1]. For E. coli, this response is triggered by expression of *rpoS* gene, whose gene product regulates the expression of about 500 downstream genes directly or indirectly [2–4]. *RpoS* mRNA is normally in a suppressed state, in which the *rpoS* mRNA 5' untranslated region (5'-UTR) Shine-Dalgarono (SD) sequence is folded inside an inhibitory stem[5, 6]. Therefore, ribosomes are prevented from binding resulting in translation inhibition on SD region so that the gene translation is turned off [1, 6]. Bacteria express small RNAs to base pair with the specific region of the *rpoS* mRNA 5′-UTR with the help of the Hfq protein when needed [7]. The inhibitory stem is opened and the SD region is released for ribosome binding [8]. Thus, it become biologically interesting to learn the spatial structure and folding dynamics of the *rpoS* mRNA inhibitory stem, which may further help us to understand Hfq-mediated sRNA-mRNA annealing process.

RNA structure and kinetics draw constant interest in gene regulation and protein translation processes [9]. However, the lack of high-resolution RNA structural data is a huge obstacle for many RNA researches. Optical tweezers are single-molecule quantitative assay, which especially suitable for studying the mechanical properties and internal interactions of long-chain macromolecules such as DNA and RNA in liquid environment [10–12]. Optical tweezers is especially suitable for studying the structure and dynamic changes of nucleic acids under tension, because it can measure single molecule conformational changes quantitatively [13]. Small angle X-ray scattering (SAXS) is a versatile and powerful technique to characterize the structure of biological macromolecules in aqueous solution in a relatively fast and convenient fashion. In this research, we measured the free energy change corresponding to the conformational change of *rpoS* RNA with optical tweezers, and dissected the RNA structure from the single molecule level. We analyzed the structure and folding/unfolding kinetics of *rpoS* mRNA inhibitory stem, by combination of optical tweezers results with overall RNA topology obtained by SARX. Through this work, we showed that the combination of optical tweezers and SAXS is a useful strategy to explore the structure-and-dynamics of RNA molecules, especially when their high-resolution three-dimensional structure are not available.

## Material and methods

### RNA preparation and setup for single-molecule stretching

The cDNA of the *rpoS* 5′-UTR was obtained by PCR amplification from Ec strain BL21 (DE3). The gene was subsequently inserted into pET-22b (+) vector (Novagen) and verified by DNA sequencing. The *rpoS* 5’-UTR with and without mutation were transcribed from DNA template as described in section 1 of S1 File. The DNA handle complementary to RNA 5’ end was a 40-nt long ssDNA oligo with a digoxigenin label at the 3’ end. The other DNA handle complementary to RNA 3’ end is a 1.1k bp long dsDNA which carries a biotin label at the 5’ end. The RNA transcript was annealed with two DNA complements in a PCR instrument. Final product molecule contains three parts: *rpoS* 5’-UTR nucleotide 466–552 which represents the core portion of *rpoS* 5′-UTR inhibitory stem, and two DNA/RNA hybrids with biotin and digoxigenin labels at the 5’ and 3’ ends, respectively.

**Fig 1:**
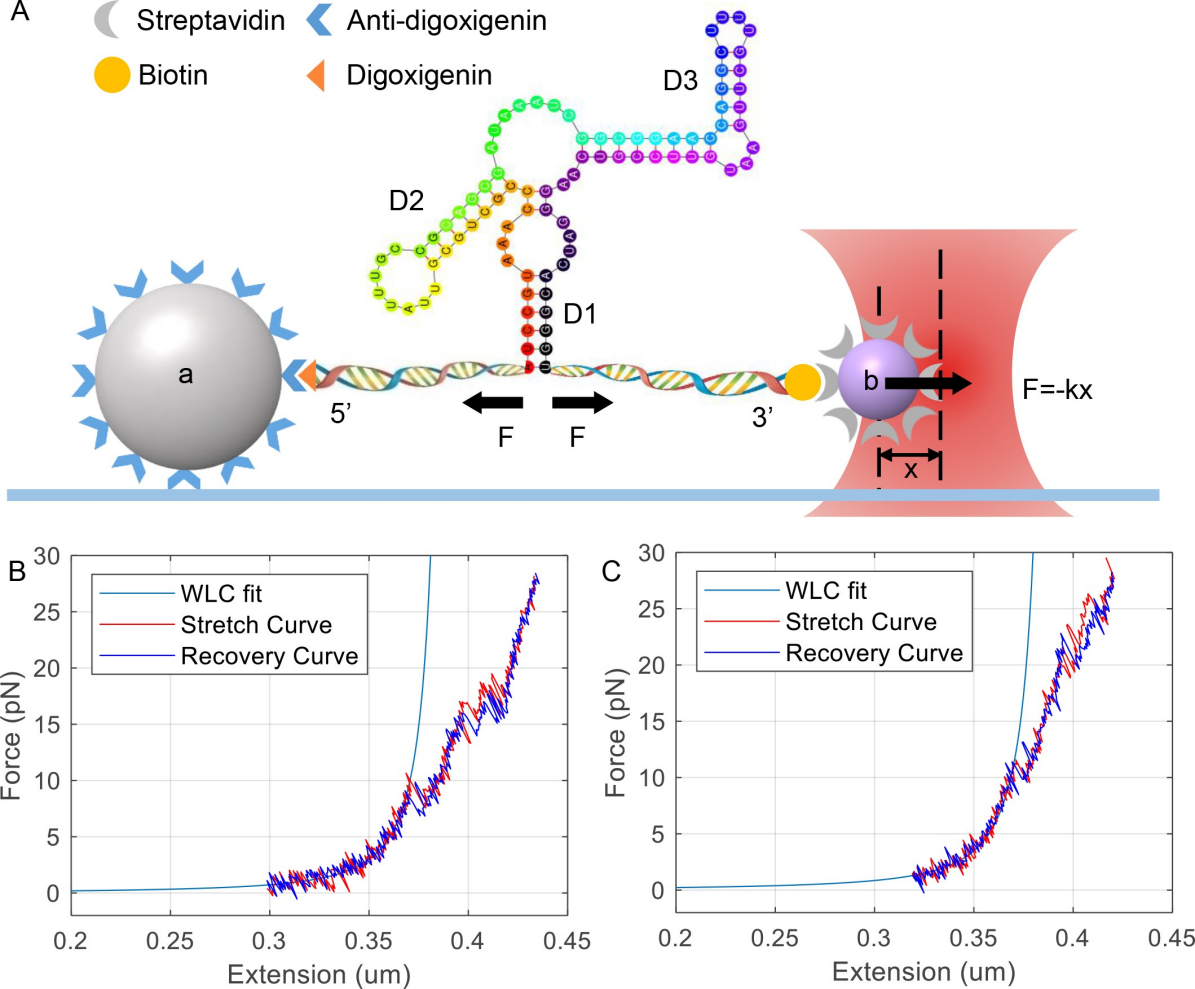
Schematic diagram of stretching *rpoS* inhibitory stem by optical tweezers and RNA force vs. extension curve (FEC). (A) 2 μm anti-digoxigenin coated polystyrene bead (a) attached to cover glass (light blue line) covalently. An 1μm streptavidin coated polystyrene bead (b) was captured by optical tweezers. The two ends of the 87-nt three-way junction (colored dot line) located between two DNA/RNA hybrid handles, that linked to a and b via digoxigenin and biotin modification. D1, D2 and D3 are three helical regions of 3-way junction. Black arrows indicate force direction. X is the distance between bead b and the center of optical tweezers. F = -Kx is the force applied on b via optical tweezers. k is stiffness of optical tweezers. (B) *rpoS* mRNA inhibitory loop unfolding (red line) /refolding (violet line) FECs without Mg^2+^. (C) unfolding/refolding FECs of same RNA in (B) but with extra 10mM MgCl_2_. The unit of X-axis is μm and of Y-axis is pN. The smooth blue curves are the fitting curves of worm-like chain model. Experimental data was acquired under a speed of 100k Hz and filtered to 100 Hz before recording.

Stretching experiment was performed under room temperature in pH 7.5 TKE buffer containing 200 mM KCl, 100 mM Tris-HCl, 1 mM EDTA and 1 ng/μl α-casein (Sigma, C6780). Optional 10 mM MgCl_2_ may be presented if otherwise noted. Optical tweezers experiment was accomplished in our “instrument for single molecule force spectrum” (S1 File Section 2). As shown in Fig 1A, the molecule was attached between an anti-digoxigenin labelled microsphere (2.11 μm diameter, Spherotech, DIGP-20-2) and a streptavidin labelled microsphere (1.07 μm, Spherotech, SVP-10-5). The 2 μm diameter microsphere was pre-attached on the chemically modified bottom of a fluidic chamber (S1 File Section 3). The single RNA molecule was stretched or relaxed by moving 1 μm microsphere with a single beam optical tweezers away or toward the 2 μm microsphere at a speed of 20 nm/s, and RNA unfolding data was obtained and recorded simultaneously. The distance (x) between 1 μm bead and the laser focus was detected by a position sensitive device (PSD). Force applied on the single molecule was calculated by multiplying x with trap stiffness k. Experimental data was acquired under a speed of 100k Hz and filtered to 100 Hz before recording. After RNA was fully extended, the motion of optical tweezers was reversed and the refolding process was recorded in the same way as unfolding process. Such process can be repeated for at most 24 times until the connection between molecule and beads broke up.

The single molecule we stretched contains three parts: *rpoS* 5’-UTR nucleotide 466–552 which represents the core portion of *rpoS* 5′-UTR inhibitory stem in the middle, and two DNA/RNA hybrids with biotin and digoxigenin labels at the 5’ and 3’ ends, respectively (S1 File Section 1). Optical tweezers experiment was performed in our “instrument for single molecule force spectrum” (S1 File Section 2). As shown in Fig 1A, the molecule was attached between an anti-digoxigenin labelled microsphere (2.11 μm diameter, Spherotech, DIGP-20-2) and a streptavidin labelled microsphere (1.07 μm, Spherotech, SVP-10-5). The 2 μm diameter microsphere was pre-attached on the chemically modified bottom of a fluidic chamber (S1 File Section 3). Stretching experiments were performed under room temperature in pH 7.5 tris-potassium-EDTA buffer (TKE, S1 File Section 3) and RNA molecules that can be repeatedly stretched were selected for analysis.

### Small angle X-ray scattering (SAXS) and its RNA sample preparation

SAXS experiments were performed at beamline BL19U2 of National Center for Protein Science Shanghai (NCPSS) at SSRF. The wavelength (λ) of X-ray radiation was set as 1.033 Å. Scattered X-ray intensities were collected using a Pilatus 1 M detector (DECTRIS Ltd). The sample-to-detector distance was set such that the detecting range of momentum transfer (q = 4p sinq/l, where 2q is the scattering angle) of SAXS experiments was 0.01–0.45 Å−1. SAXS data were collected as 20 × 1 s exposures and scattering profiles for 20 passes were compared at 20°C using 60 μl sample in 50 mM Tris-HCl, pH 7.0, 150 mM NaCl. Measurements were carried out at two different concentrations of 2 and 4 mg/ml. The data were analyzed in the ATSAS package [14] following the standard procedures. After subtracting buffer scattering, the data curves from different concentrations were scaled and merged using PRIMUS [15]. GNOM [16] was employed for estimating the particle maximum dimension (Dmax), real space Rg of the RNA, and calculation of the pair distance distribution function (PDDF). The ab initio envelopes of the folded *rpoS* RNAs were determined using DAMMIN [17] with 20 runs for each experimental group. DAMAVER was used to analyze the normalized spatial discrepancy between the 20 models. The filtered SAXS model by DAMFILT was showed in VMD. Linear Guinier plots in the Guinier region (q × Rg<1.3) were confirmed (S1 File Section 4, Fig F-A).

RNA sample for SAXS was prepared by in-vitro transcription. The DNA template for transcription of *rpoS* 5′-UTR nucleotide 466–552 capped with two 5′-guanine and two 3′-cytosine was prepared by PCR amplification from the target plasmid using a forward (GAAATTAATACGACTCACTATAGGATCCGTAAACCCGCTGCGTTATTTCG) and a reverse (GGACCCGTGATCCCTTGACGGAACATTCAAGCAAA) primers. The in-vitro transcription mixture contained DNA template, 40 mM Tris, 10 mM DTT, 5 mM NTPs, 40 mM MgCl2, 1 mM spermidine, 0.01% (v/v) Triton X-100, 1 mg T7 RNA polymerase. The reaction was incubated in a water bath at 37°C for 4 hours. The transcription product was precipitated with ethanol at -20°C overnight, then dissolved in DEPC-treated water. The 91-nt RNA sample was separated by electrophoresis on urea-containing polyacrylamide denaturing gels and purified by Elutrap Electroelution System (GE Healthcare). Final RNA product was dialyzed into an SAXS buffer (10 mM NaH2PO4, 50 mM NaCl, pH 6.5) and quantified by absorbance at 260 nm.

## Results and discussion

The secondary structure of *rpoS* mRNA inhibitory stem was previously reported as a three way junction [5]. The prediction using Mfold website (http://unafold.rna.albany.edu/?q=mfold) also gave a same structure (Fig 1A) [18]. We first pulled apart single RNA molecule using optical tweezers to obtain the force vs. extension curve (FEC). The single RNA molecule was stretched or relaxed by moving 1 μm microsphere with a single beam optical tweezers away or toward the 2 μm microsphere (Fig 1A). FECs of RNA unfolding/refolding are recorded (Fig 1B and 1C). The saw tooth breaks on FEC indicated a part of RNA inhibitory stem was unfolded/refolded here. After repeated testings, we found that FECs contain the most rips when the stretching speed was 20 nm/s (data not shown). Therefore, we used this speed for all stretching experiments in this research.

**Fig 2:**
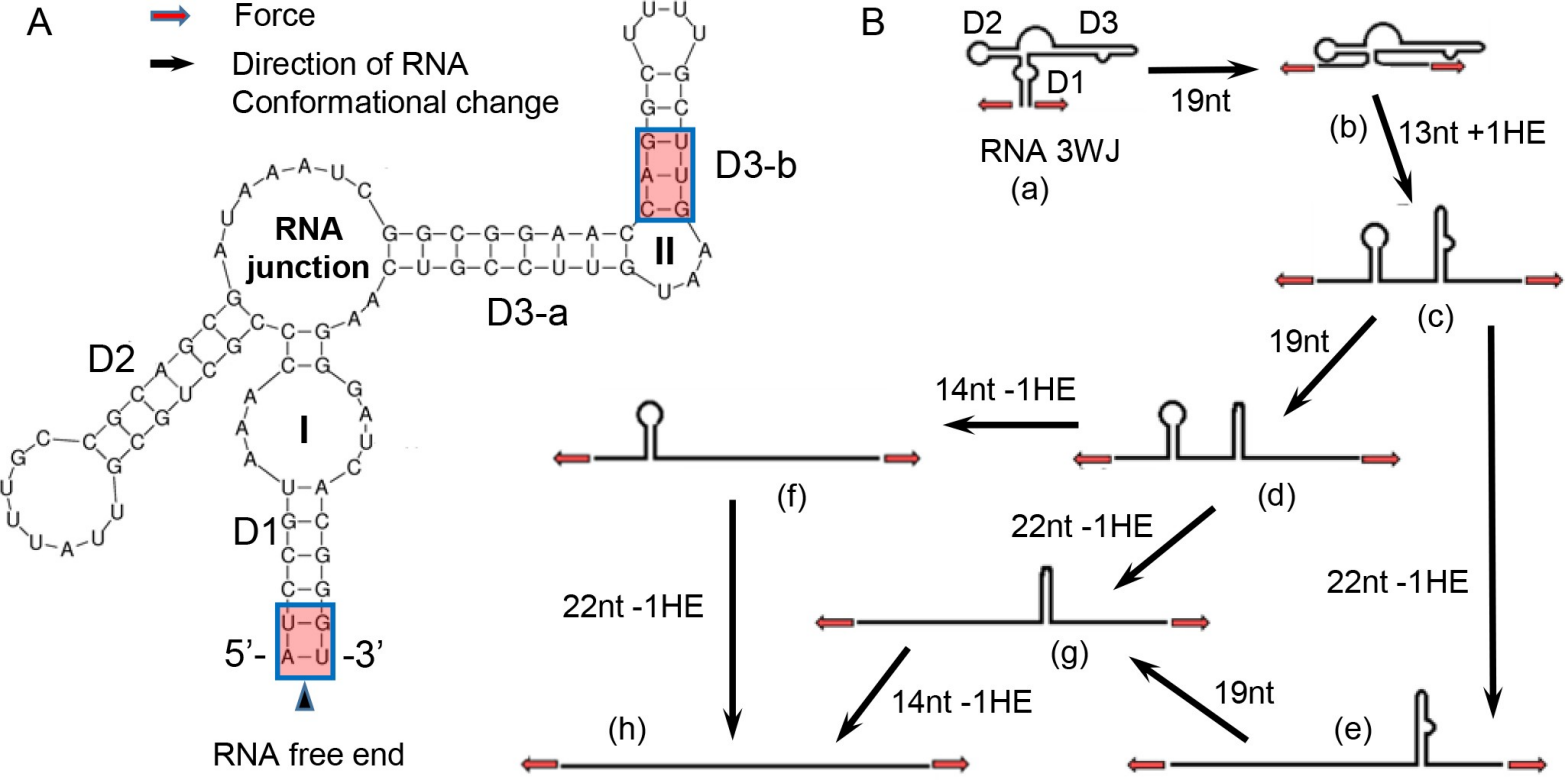
Secondary structures of *rpoS* inhibitory stem and its conformational changes under external force. (A) the secondary structures of *rpoS* inhibitory stem. Roman numerals I, II indicate different RNA bulges. Inside red boxes are weak internal cohesion region. (B) RNA intermediate states and potential pathway of conformational changes. The intermediate states are labelled from a to h. Numbers beside black arrows are the number of nucleotides (followed by nt) that are released during a state change in the direction of the arrow, and number of helix ends (HE) that are exposed (+) or eliminated (-) during RNA unfolding.

The secondary structure of *rpoS* inhibitory stem and all potential RNA conformational changes were summarized in Fig 2 to analyze the relationship between each FECs’ rip event and RNA subunits predicted from RNA secondary structure. Fig 2A displayed five relatively independent subunit of predicted inhibitory stem secondary structure: double helix D1, D2, D3-a, D3-b and the RNA junction. The potential intermediate states which RNA most likely stay during unfolding/refolding locate right behind two bulges (bulge I and II) and the major RNA junction (Fig 2B, from a to h). Notably, more than one subunit may be unfolded or refolded in one rip simultaneously. Thus, the molecule may jump over one or more intermediate states.

In order to analyze unfolding and refolding pathway at the same time, we re-aligned all identified rips in a sequence from folding state to unfolding state. RNA rip lengths were measured from FECs (Fig D). Expected RNA extensions were calculated with worm-like-chain model according to changes in number of nucleotides and helix ends in Fig 2B (S1 File Section 4). After matching each rip length with the secondary structure change in Fig 2A, most of magnesium-free curves could be classified into two groups (S1 File Section 4 and Table A). The first group stopped at intermediate states c and f in Fig 2B, during unfolding pathway a→c→f→h. The subunits that opened during each rip were: D1+RNA junction, D3, and D2. The second group followed the pathway a→c→d→h with the subunits opened in a sequence of D1+RNA junction, D3-a, and D3-b+D2.

In order to validate our interpretation, we stretched RNA molecules without D2 region. Rip lengths measured from some FECs indicated D1, RNA junction, D3-a and D3-b were opened in an order which is consistent with the secondary structure predicted by Mfold. Sometimes D1 and RNA junction were opened simultaneously. Such simultaneous opening also occurred on D3-a and D3-b occasionally (Fig E).

Magnesium ions are often found to play a crucial role in stabilizing RNA tertiary folding. To learn the impacts of Mg^2+^ binding on the conformation of *rpoS* inhibitory stem, the RNA was again stretched in the presence of excess Mg^2+^ to investigate solution structure of inhibitory stem. In fact, rip lengths in the presence of Mg^2+^ is highly consistent with the length of the secondary structure model predicted under no Mg^2+^ condition. For most of FECs that contain three rips, favored unfolding pathways were divided into three groups: a→c→f→h, a→c→e→h and a→c→d→h (Fig 2B and Table B), when magnesium ions were present in the solution. Table 1 showed a perfect matched example of one FEC containing four rips. The first rip takes place at 9.16 pN with a 7.38 nm rip length, which matches the length of D1 opening event (7.45 nm long according to the RNA secondary structure). The second rip is consistent with the opening of core junction very well (7.01 nm *vs*. 7.30 nm). The third rip has a rip length of 8.12 nm; which is close to the opening length of D3-a, while the fourth rip length is 11.07 nm long which matches the simultaneous opening of D2 and D3-b (11.93 nm according to the RNA secondary structure). In general, the RNA extension is fitted nicely with the secondary structure prediction.

**Table 1:** Example of RNA rip force and length with 10mM MgCl_2_.

	Force (pN)	Rip length (nm): experimental/expected
First rip	9.16	7.38 /7.45
Second rip	10.96	7.01/7.30
Third rip	17.55	8.12/8.49
Fourth rip	18.08	11.07/11.93

Sequence of unfolded RNA subunits: D1; core junction; D3-a; D2 + D3-b, numbers in bold are expected rip length calculated from Fig 2.

FECs with and without magnesium ion both agreed with the intermediate states of predicted RNA secondary structure, which suggests *rpoS* inhibitory stem maintains same secondary structure arrangement in the absence and presence of Mg^2+^. We further suspected whether Mg^2+^ binding may affect the free energy stored inside the inhibitory stem. A theory developed by Gavin Crooks [19] illustrated that the free energy can be calculated from the analysis by optical tweezers during RNA unfolding/refolding (S1 File Section 5). Therefore, rip events corresponding to three RNA subunits (f→h for D2, c→d and e→g for D3-a, b→c for RNA junction) were picked out from FECs (Table A&B) for further analysis. The free energies of three RNA subunits (D2, D3-a, RNA junction) were calculated from optical tweezer measurements, in the absence and presence of Mg^2+^, and listed in Table 2. Free energies of D2 and D3-a without Mg^2+^ were also calculated with Mfold software (37 degree, 1M NaCl). From Table 2, one can noticed that free energy values calculated from FECs agreed with Mfold prediction within a ~10% difference.

**Table 2:** Free energy of RNA subunits.

	Rip force (pN)	Rip length (nm)	ΔG (kJ/mole)	ΔG_Mfold_(kJ/mole)
D2	16.71±1.26	8.92±0.85	57.1	51.19
D3-a	11.74±0.49	8.86±1.61	48.3	51.61
RNA junction	6.62±1.39	7.01±0.79	18.9	N/A
D2 (10mM MgCl_2_)	24.15±2.28	7.56±0.68	62.9	N/A
D3-a (10mM MgCl_2_)	22.36±3.3	8.22±1.62	66.9	N/A
RNA junction (10mM MgCl_2_)	10.83±2.19	7.71±0.74	34.9	N/A

With the addition of 10 mM of MgCl_2_, the free energy of RNA junction increased from 18.9 to 34.9 kJ/mole. The significantly increased free energy of all RNA subunits due to the presence of Mg^2+^ suggested that magnesium ions dramatically enhanced the internal interaction of *rpoS* inhibitory stem. Noticing that the stem orientation was mostly determined by RNA junction [20], we hypothesized Mg^2+^ might compressed RNA junction with reduced free energy. Compressed RNA junction rearrange the relative orientation of different stems of inhibitory three-way junction.

**Fig 3:**
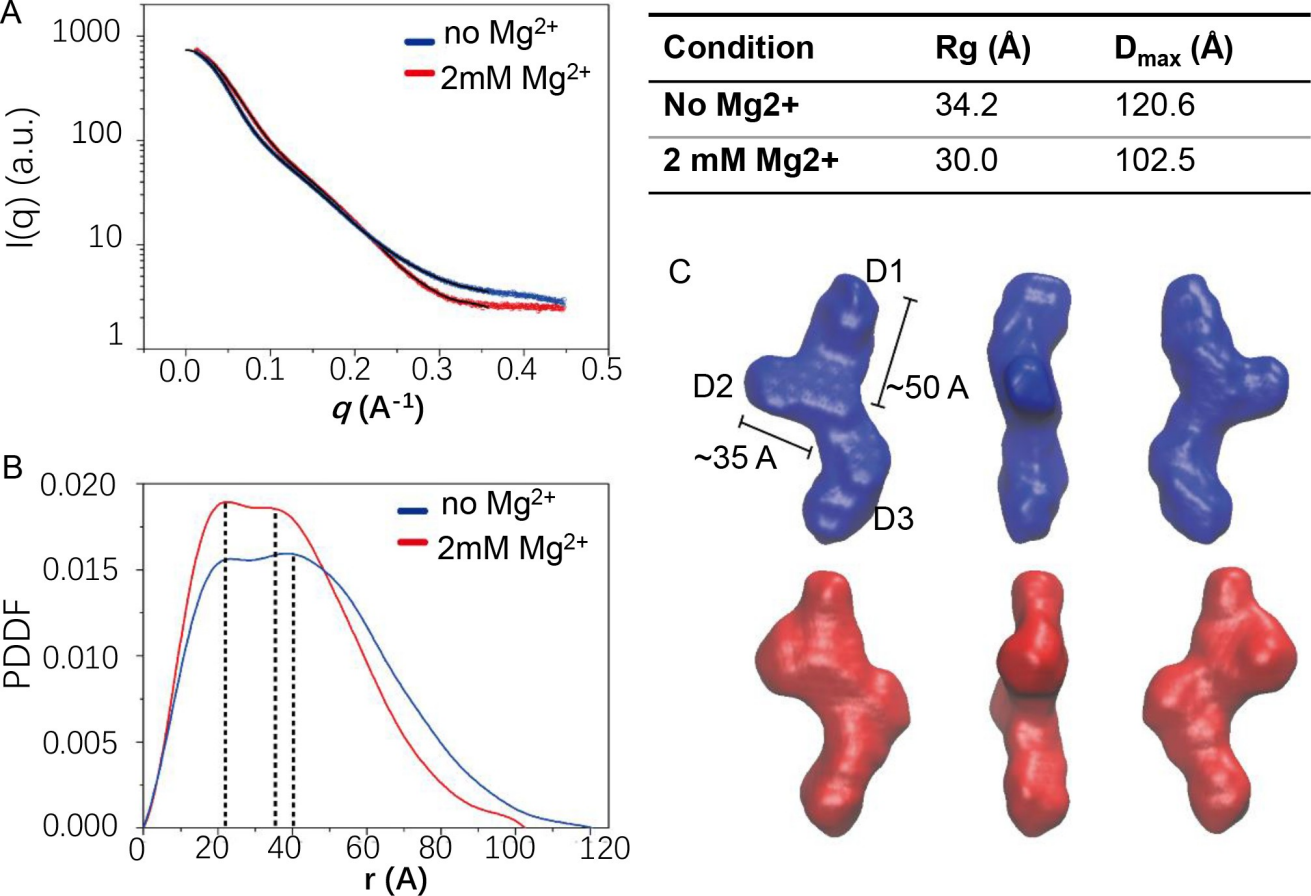
Small angle X-ray scattering (SAXS) analysis. SAXS indicates a tertiary folding of *rpoS* inhibitory stem as a three-way junction and Mg^2+^ binding induces its conformational transition from a relatively relaxed structure to a compact one. (A) 1D scattering curves of *rpoS* inhibitory stem in the absence (blue) and presence (red) of 2 mM Mg^2+^. (B) The pair distance distribution function (PDDF) for 1D SAXS curves in (A). (C) 3D envelopes of inhibitory stem with (red) and without (blue) 2 mM Mg^2+^ calculated using DAMMIN.

Therefore, we employed SAXS to investigate the structure of a 91-nt *rpoS* inhibitory stem of the RNA sample by visualizing the overall topology of RNA inhibitory stem (S1 File Section 6). The SAXS profiles summarized in Fig 3 showed that Real space Rg values of inhibitory stem is 34.2 Å while its D_max_ value is 120.6 Å. The ab initio shape model of inhibitory stem calculated by DAMMIN shows a non-symmetric “Y”-shape topology (Fig 3C), confirming the generation of a three-way junction folding of inhibitory stem in solution. This “Y”-shape architecture has two upper arms with different length (~35 and ~50 Å), basically consistent with the 7 base-pair stem-loop (D2) and 10 base-pair stem (D1) which also contains a non-symmetric internal loop. The longest non-straight arm is tentatively assigned as the 13 base-pair stem-loop structure with a 3-nucleotide bulge (D3).

Upon the addition of 2 mM Mg^2+^, the values of R_g_ and D_max_ decreased to 30.0 Å and 102.5 Å (Fig 3. Inset table), respectively, exhibiting compaction for the overall structure of inhibitory stem. This is consistent with the Kratky profiles shown in Fig F. Intriguingly, the superimposition of inhibitory stem shape models in the absence and presence of 2 mM Mg^2+^ reveal an obvious inhibitory stem conformational change upon the binding with Mg^2+^ ion. Conformational changes is more obvious where D1 is shifted towards D2 with an ~30° rotation of the helix axis from its position of inhibitory stem without Mg^2+^ (S1 File Section 6 Fig F-C). An extra envelope density, close to the junction region, is observed for inhibitory stem shape model in the presence of Mg^2+^. This could be the consequence of the re-orientation of D1 or a local structural rearrangement in the core junction because of Mg^2+^ binding. Mg^2+^ binding might produce new base pairing in single-stranded nucleotides that link different stems. In addition, the SAXS p(r) plots of inhibitory stem with and without Mg^2+^ both contain two peaks (Fig 3B), corresponding to two most populated distances in the structure of inhibitory stem. The peak at ~22Å shared by both plots is a typical symbol for A-form helical width as suggested by previous researches [21, 22]. Another two peaks are located at ~40 Å and ~35 Å, respectively, in p(r) plots of inhibitory stem in the absence and presence of Mg^2+^. This suggests that the distance between two major structural elements of *rpoS* inhibitory stem is changed upon binding with Mg^2+^, which is consistent with our observation of a Mg^2+^-induced re-orientation of D2.

Peng et al. previously indicated that Hfq folds the *rpoS* mRNA leader into a specific tertiary structure via multiple interaction sites on *rpoS*, including a classic (AAN)_4_ motif located upstream of the three-way junction studied here and a “U_5_” loop motif capping D2. Hfq binding with “U_5_” loop motif partially open the inhibitory stem to facilitate the sRNA annealing and ribosome binding [6]. In this research, we first proposed the hypothesis that Mg^2+^ reoriented RNA stem by reforming RNA junction through optical tweezer experiments and SAXS topology study later. The SD sequence of *rpoS* 5’-UTR is immediately adjacent to the D1. Mg^2+^-binding reduced the distance between D2 U_5_ site and SD sequence. The shorter distance may improve Hfq-bridged annealing between sRNA and *rpoS*. Idealy, our strategy of combining optical tweezers and SAXS can be further expanded to the investigation of Hfq-*rpoS* RNA interaction and may pave a way towards better understanding of how RNA stability was affected by different ligands.

RNA structure determination is highly challenging given the facts that RNA molecules are usually difficult for crystallization and molecular weight limitation of solution nuclear magnetic resonance (NMR) for RNA samples. SAXS has been widely used to investigate the overall conformation of biological macromolecules in the recent decade. It is particular useful in defining the topology structure of folded RNA molecule due to strong electron density along phosphate-sugar backbones [23]. Optical tweezers, on the other hand, have been long recognized as an efficient single-molecule technique in investigating RNA structure and folding dynamics. Therefore, optical tweezers and SAXS can work collaboratively to explore the structure-and-dynamics relationship of RNA molecule, especially when their high-resolution three-dimensional structure is not available. In addition, numerous RNAs can fold into multiple structures to be functional [24]. Given the ability to unfold/refold one single molecule using optical tweezers, the approach to combine single-molecule measurement technique and low-resolution structural biology method in this research may provide us a new and convenient route to analyze and characterize different RNA folding structures.

## Supporting information

S1 File Supporting material, data table and data analysis methods. Description of sample preparation, optical tweezers setup, methods used in data analyzing and table of rip lengths.
(DOCX)
