# Supplementary material for: Conformation and mechanical property of rpoS mRNA inhibitory stem studied by optical tweezers and X-ray scattering
Source: PLoS One. 2019 Sep 26;14(9):e0222938. doi: 10.1371/journal.pone.0222938 (PMC6762075; doi:10.1371/journal.pone.0222938)
Supplement: S1 File — Description of sample preparation, optical tweezers setup, methods used in data analyzing and table of rip lengths. (DOCX) [file pone.0222938.s001.docx]

Supporting material, data and data analysis methods

1. **Preparation of RNA sample for optical tweezers**

**1.1** **Method and process for preparing template DNA**

The *rpoS* 5′-UTR was obtained by PCR amplification from Ec strain BL21 (DE3). The gene was subsequently inserted into pET-22b (+) vector (Novagen) and verified by DNA sequencing (Fig. A- A). Linear template DNA (Fig.A- B) was amplified from the resulting plasmids with template forward primer and reverse primer. The PCR template forward primers (GAAATTAATACGACTCACTATACAGTTCAACACGCTTGCATTTTGAAATTCGTTACAAGGGG) located 40bp upstream of *rpoS* sequence with an extra sequence of T7 promoter at 5' end of the primer (Underlined part). The template reveres primer (TTATTGCTCAGCGGTGGCAGCAGCCAACTCAGCTTCCTTTCGGGC) located at the 5′ end of UTR sequence. That gave an 1246bp dsDNA with T7 promoter sequence upstream to *rpoS* 5’-UTR sequence (Fig.A- B).


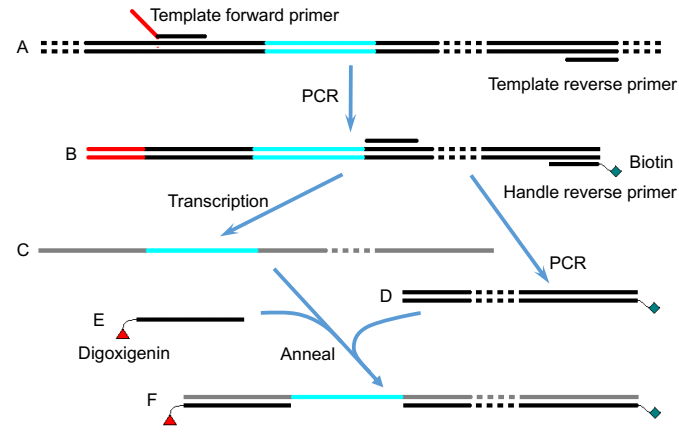


**Fig A. The procedure of RNA sample preparation.** Black lines denoted DNA, gray lines denoted RNA, red lines denoted T7 promoter sequence, cyan lines denoted sequence of *rpoS* RNA inhibitory stem, and arrows illustrate the direction of reaction. (A) represents the plasmid containing *rpoS* sequences; (B) represents the template DNA obtained from plasmid amplification; (C) represents the full-length *rpoS* 5'-UTR RNA transcribed from template; (D) represents the right handle; E represents the left handle; F represents the final product for optical tweezers experiment.

**1.2** **RNA transcription**

*RpoS* RNA 5′-UTR was transcribed from the template DNA (Fig.A- B) using standard RNA transcription kit (Ambion, MEGAscript T7, AM1334), the resulting 1224nt single stranded RNA containing 87nt inhibitory stem in the middle. Gel electrophoresis diagram showed that the product presented a single band (Fig.B).


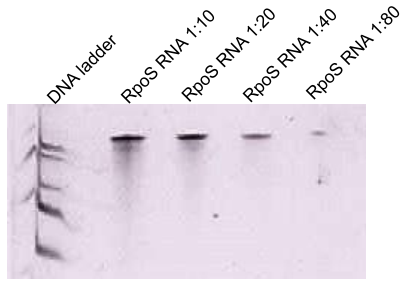


**Fig B. *RpoS* RNA 5’-UTR Gel electrophoresis diagram.** From left to right were DNA Ladder, 10-fold diluted, 20-fold diluted, 40-fold diluted, and 80-fold diluted *rpoS* RNA, respectively.

**1.3 Preparation of DNA right handle**

Most of transcribed *rpoS* RNA 5’-UTR, except the 87 nt inhibitory stem, were covered by DNA complementary to form DNA/RNA hybrid double helix as the stretched handle. DNA complementary of right side (Fig A- D) was amplified through PCR using DNA template (Fig A- B), handle forward primer and handle reverse primer. The 5' end of handle reverse primer was labeled with biotin to enable bead connection for final product. The primer sequence is:

Handle forward primer：AGGAGCCACCUUAUGAGUCAGAAUACGCUGAAAGUUCAUG

Handle reverse primer: biotin-TTATTGCTCAGCGGTGGCAGCAGCCAACTCAGCTTCCTTTCGGGC

**1.4 RNA/DNA handle annealing**

Finally, full length RNA (Fig A- C) was mixed with synthetic 3’ digoxigenin labeled DNA oligo (dig-oligo, Fig A- E) and the right handle (Fig A- D) inside the annealing buffer(80% formamide (Ambion), 1mM EDTA pH 8.0, 40mM Pipes pH 6.3, 0.4M NaCl), in a mole ratio of 1:1:2. The 3' biotinylated 40 base pairs long left handle is commercial available. Therefore, the complicate 3' labeling process is bypassed. The annealing was performed as following: 85˚C for 10 minutes, 62˚C for 1.5 hours , 52˚C for 1.5 hours, and then ramp to 10˚C in 10 minutes. Obtained mixture was purified by ethanol precipitation. The final sample (Fig A- F) carried biotin and digoxin at two ends, and the unmatched portion in the middle was the RNA inhibitory stem that would be studied later.

Dig-oligo sequence：CCCCTTGTAACGAATTTCAAAATGCAAGCGTGTTGAACTG-dig

**2. Instrument for single molecule force spectrum**

The RNA stretching strategy was to put RNA molecule between of two micro-spheres via antibody-antigen interaction. The 2μm-diameter micro-spheres was fixed on the bottom of the fluidic chamber. The 1μm-diameter micro-spheres was manipulated by optical tweezers for RNA stretching. A Labview (National Instrument) program written in our lab was used to implement feedback control, automated stretching, data acquisition/filtration, and real-time display of stretching curves. Fig C shows the schematic drawing of experimental setup.


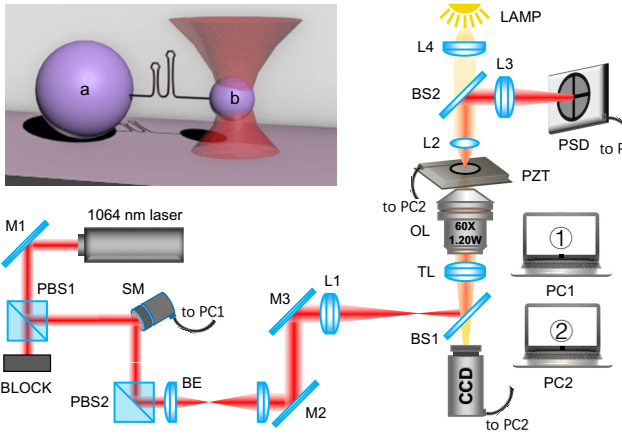


**Fig C. Illustration of optical tweezers setup for RNA stretching experiment.** The upper-left insertion was a schematic drawing of experiment: the larger micro-sphere was fixed to the surface of the chemically modified cover glass. The stretched RNA molecule was connected between the larger micro-sphere and a freely floating smaller micro-sphere. M1-3 are 1064 nm total reflection coated mirror. PBS1&2 are polarizing beam splitters. PZT is a piezoelectric nano-stage. SM is a piezoelectric steering mirror. BE is a 1.5 times beam expander. L1, L3, L4 are lens. BS1-2 are dichroic mirror. TL is the tube lens. OL is 60X objective lens; L2 is the condenser. PC1-2 are computers and PSD represents position sensitive detector.

**2.1** **Design of optical tweezers measurement system**

The 1064 nm fiber laser (Amonics, AFL-1064-40-R-CL) is reflected by M1 and transformed into linearly polarized beam by PBS. Exiting beam is reflected into beam expander by steering mirror (PI, S-330.2SL) and another PBS and a piezoelectric steering mirror employed to adjust the position of the optical tweezers. The expanded laser beam is then reflected by M2, M3 and BS1, coupled to a microscope (OLYMPUS, IX 71), and focused onto the sample by an objective lens (OLYMPUS, uPlanSAPO 60X, N.A. 1.20). In the experiment, the fluidic chamber was placed on the top of PZT stage (PI, P-545.3R7). A fixed reference micro-sphere was imaged on the CCD through the objective lens. The image of the reference micro-sphere collected by CCD is used to calculate three-dimensional drifting of the micro stage. The position is transmitted into computer 2 through RS232 interface. Computer 2 controls the movement of PZT stage to cancel the influence of the mechanical drift of the micro stage. Computer 2 is also used for signal acquisition and driving the steering mirror to implement RNA stretching experiment.

During experiments, the piezoelectric steering mirror controls the optical tweezers to manipulate the smaller micro-sphere away from the larger micro-sphere in order to stretch the RNA. The laser passed through smaller micro-sphere was collected by a set of lens and captured by the position sensitive detector (PSD, Pacific Silicon Sensor, DL100-7PCBA3) locating at the back focal plane. The detector acquired deviation signal of smaller micro-sphere from the center of the optical trap, with an accuracy less than 1 nm. A Labview program written in our lab was employed to implement all the functions of device control, signal feedback, and data acquisition. The stiffness K of the optical tweezers system was calculated from collected power spectrum data. The pull force applied on the *rpoS* RNA molecule is calculated by F = - KX with an accuracy of 0.5 pN.

**2.2** **Optical tweezers stretching experiment:**

In experiment, a floating smaller micro-sphere was trapped by optical tweezers and manipulated toward a larger micro-sphere fixed on the bottom repeatedly until a connection was established. At this point, the end with digoxigenin of the RNA was connected to the larger micro-sphere modified with the Digoxigenin antibody (the larger micro-sphere was attached to the bottom of the sample chamber). The other end of the RNA was linked to the streptavidin-modified micro-spheres by the specific coupling of biotin with streptavidin. The floating smaller micro-spheres were trapped and moved away from the fixed larger micro-spheres by optical tweezers to stretch the RNA. The distance (x) between 1 μm bead and the laser focus was detected by a position sensitive device (PSD). Force applied on the single molecule was calculated by multiplying x with trap stiffness k. Experimental data was acquired under a speed of 100k Hz and filtered to 100 Hz before recording.

Once the force applied on RNA molecule increased, the DNA/RNA hybrid handle gradually elongated. The obtained force *vs.* extension curve (FEC) could be well described by worm-like chain model. When the force increased to certain level, part of RNA structure was disrupted and a small RNA fragment was released from folded RNA molecule. Abrupt increase in RNA extension introduced a saw-tooth shape rip on the RNA FEC. After RNA was fully extended, the motion of optical tweezers were reversed and the refolding process was recorded in the same way as unfolding process. Such process can be repeated for at most 24 times until the connection between molecule and beads broke up.

**3. Chemically modified cover glass and preparation of sample chamber**

In our RNA stretching experiment, 2-micrometer-diameter micro-spheres were fixed on the bottom of the fluidic chamber. To guarantee the solidity of micro-spheres binding during optical tweezers experiment, micro-spheres were fixed on chemical modified surface via covalent bonds. The process of cover glass preparation is based on the work of Matthew P. Nicholas, Lu Rao, and Arne Gennerich with minor modifications [1]. First, the cover glass (CORNING, 2845-22) was scrub with alcohol, and sonicated for 5 minutes in an 0.2M potassium hydroxide solution. The cover glass was rinsed repeatedly with deionized water, and dried for 30 minutes at 120 degree inside oven. After drying, the cover glass was cleaned with plasma under 800 μtor argon and oxygen atmosphere for 2-3 minutes. Plasma-cleaned cover glass was placed in air for 15 minutes, then immersed in pure acetone, acetone containing 2% (v/v) APTES in sequence for 15-30 seconds each time. After that, the cover glass were put in and out of acetone for a couple of times, and then placed into 120 degree oven for 30 minutes. Then the cover glass was cooled in air, washed repeatedly with alcohol and deionized water, and dried after ultrasonic treatment. Finally, the treated cover glass was stored in vacuum desiccator for future use.

Before optical tweezers experiment, two 25 mm x 5 mm parafilm strips (Cole-Parmer, PM-996) were placed on the middle of a glass slide, long edge parallel, 15-20 mm apart face to face. Then a chemically modified cover glass was put on top of the two parafilms. The whole architecture was put onto a 70 degree hot plat to melt the parafilms. The architecture was gently pressing to ensure a reliable sealing before cool down. Finally, a 100 μm thick channel between the two parafilms was established as the sample chamber. The chamber volume was about 20 microliter.

The fluidic chamber was incubated with 8% glutaraldehyde for 30 minutes at room temperature, washed with 400 ul deionized water and blew to dry with compressed air. The 2 μm diameter anti-digoxigenin coated micro-spheres (Spherotech, DIGP-20-2, 2.11um) was washed with PBS (Thermo, SH30256.0113) buffer before deployed into the fluidic chamber. The resulted 30 μl micro-spheres solution was injected into the fluidic chamber and incubated at room temperature for 30 minutes to ensure firmly attachment.

The fluidic chamber was then washed with 400ul TKE buffer (200 mM KCl, 100mM Tris-HCl, 1mM EDTA, and 1ng/ul α-casein (Sigma, C6780 α-Casein from bovine milk), pH 7.5, optional 10mM MgCl_2_ may presents if noted) and incubated again at room temperature for 30 minutes to block the unconnected covalent bonds.

1μm diameter streptavidin-labeled micro-spheres (Spherotech, SVP-10-5, 1.07um) were washed with PBS and TKE buffer in sequence. Then microspheres were diluted 50 times and mixed with 1 ul 200-times-diluted RNA solution, kept rotating in 4 degree for 120 minutes. After incubation, the RNA-bond micro-spheres were injected into the TKE washed fluidic chamber for experiment.

**4. RNA rip length measurement**

RNA disruption introduced a sudden increase of its end-to-end distance and the tension along the molecule decreased. Therefore, the force vs. extension curve turned downward at point A in Fig D. In response to tension decrease, extension of DNA / RNA hybrid handles at both ends of the RNA molecule shrink so that the length changes before and after the jump were smaller than the length of RNA extension (point B in Fig D). Once the force increased to the same level of the begin (point C in Fig D), the length of the DNA handle became the same as begin. Therefore, the real length of the newly opened ssRNA at this tension force was equal to the distance between point A and C. If a second rip occurs before the external force rises to the begin, the middle part of the two jumps could be extended to determine the position of the reference point, as shown the point C in Fig D in the measurement of the first open length.


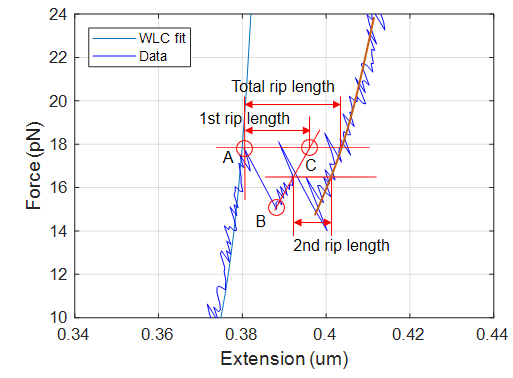


**Fig D. RNA opening length measurement method schematic diagram.** The blue curve is the experimental data and the cyan curve is the result of worm-like-chain (WLC) model fitting result. The red arrow line is the length of opening RNA structure. A, B, C identify the measuring points, the red circles are the characteristic positions of the RNA structural changes.

The distance between 5′ and 3′ ends of RNA consists of ssRNA and free helix ends. During each transition, the changes of single-stranded nucleotide and helix ends are all indicated by the numbers in Fig 2B. RNA in solution exists mostly as A-form helix with a ~2 nm diameter. Therefore, exposing or eliminating one helix end will introduce a 2 nm increase/decrease in RNA extension. Liphardt et.al [2] illustrated that the extension of each free nucleotide can be calculated using the worm-like chain model [3]:

$\frac{FP_{l}}{k_{B}T}=\frac{1}{4{(1-\frac{x}{L})}^{2}}+\frac{x}{L}-\frac{1}{4}$ (1)

Where F is the force applied by tweezers; Pl represents the persistence length; kB is the Boltzmann constant; T is temperature; L equal to 0.59 nm which is the distance between two adjacent phosphorous atoms on RNA backbone; x represents the RNA extension added by releasing one nucleotide.

**Table A. Classification of *rpoS* RNA inhibitory stem rip sequence without Mg^2+^**

| **Open** | **First rip** | | **Second rip** | | **Third rip** | |
| --- | --- | --- | --- | --- | --- | --- |
| Way | Force(pN) | length(nm)  Experiment / **theory** | Force(pN) | length(nm)  Experiment / **theory** | Force(pN) | length(nm)  Experiment / theory |
| a🡪c🡪d🡪h | 7.01±1.80 | 12.24±0.99 | 12.62±1.63 | 9.36±0.91 | 16.36±1.23 | 12.74±1.34 |
|  |  | **13.56** |  | **8.13** |  | **11.18** |
| a🡪c🡪f🡪h | 8.63±1.38 | 12.54±1.48 | 15.83±1.48 | 11.88±1.15 | 16.71±1.26 | 8.92±0.85 |
|  |  | **13.56** |  | **11.92** |  | **7.39** |
| b🡪c🡪d🡪h | 7.14±1.10 | 6.64±0.37 | 11.74±0.49 | 8.86±1.61 | 15.31±1.76 | 12.55±1.61 |
|  |  | **6.82** |  | **8.13** |  | **11.18** |
| b🡪c🡪e🡪g | 5.03 | 8.11 | 5.93 | 8.12 | 13.77 | 8.49 |
|  |  | **6.82** |  | **7.22** |  | **8.13** |

Bold type is the expected rip length calculated from the secondary structure predicted by Mfold software.

Rips corresponding to transition between state c and d had relatively big extension comparing to the secondary structure prediction (9.36 nm vs. 8.13 nm). This could be explained by the weak base-paring in the portion of D3-b adjacent to bulge II, which might open together with D3-a.

There were also two relatively rare cases in which D1 was unfolded before the first rip (Fig 2B, b🡪c🡪d🡪h and b🡪c🡪e—g). Given the facts that the base-pairing inside D1 was relatively weak (Fig 2A), this scenario might be explained by an inherent instability relating to D1 region of rpoS inhibitory stem. In conclusion, the consistency between rip lengths and the RNA secondary structure indicated the rpoS inhibitory stem very likely folds as the secondary structural model predicted by Mfold.

Mutated RNAs without D2 region were stretched to validate our interpretation. Fig E illustrated the secondary structure of mutated RNA predicted by Mfold and its conformational changes under external forces. After matching rip lengths measured from FECs with expected RNA open lengths, most of RNA rips were classified as a transition of opening a specific RNA structure (Fig E- B). When an external force was applied on the two free ends of RNA, D1, RNA junction, D3-a and D3-b were opened in order. Sometimes D1 and RNA junction, or D3-a and D3-b were opened simultaneously.


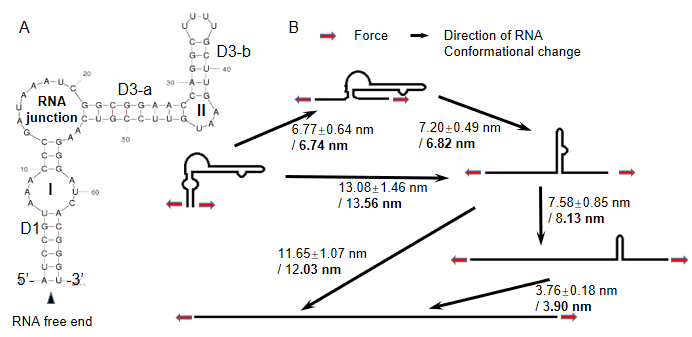


**Fig E. Secondary structures of RNA without D2 region and its conformational changes under external force.** (A) the secondary structures of rpoS inhibitory stem without D2 region. Roman numerals I, II indicate different RNA bulges. (B) RNA intermediate states and potential pathway of conformational changes. Numbers beside black arrows are the rip lengths measured from FECs. Bold numbers are expected rip lengths calculated from the secondary structure.

FECs in present of Mg^2+^ were grouped in Table B. Most of FECs started from microstate a and unfolded to microstate h. Rips corresponding to transition between state a and c had relatively short extension compared with the secondary structure prediction (11.33 nm vs. 14.74 nm). The force needed for the first rip increased over 40% in the presence of Mg^2+^. Therefore, the reduced RNA extension could be attributed to the open end of D1 helix containing a AU and a GU base pairs. The pair is easy to unfold before first detectable rip under certain circumstances.

**Table B. Classification of *rpoS* RNA inhibitory stem rip sequence with Mg^2+^**

| **Open** | **First rip** | | **Second rip** | | **Third rip** | |
| --- | --- | --- | --- | --- | --- | --- |
| Way | Force(pN) | length(nm)  Experiment / **theory** | Force(pN) | length(nm)  Experiment / **theory** | Force(pN) | length(nm)  Experiment / theory |
| a🡪c🡪e🡪h | 11.68±1.02 | 14.02±0.52 | 17.04±0.55 | 8.30±1.30 | 18.89±1.65 | 12.92±0.52 |
|  |  | **14.74** |  | **7.63** |  | **12.60** |
| a🡪c🡪f🡪h | 11.75±1.66 | 11.33±1.78 | 20.45±2.36 | 11.34±1.00 | 24.15±2.28 | 7.56±0.68 |
|  |  | **14.74** |  | **12.54** |  | **7.88** |
| a🡪c🡪d🡪h | 12.34±0.90 | 10.94±2.10 | 20.44±1.62 | 9.10±1.70 | 23.95±3.89 | 11.56±0.93 |
|  |  | 14.74 |  | 8.49 |  | 11.93 |
| b🡪c🡪f🡪h | 10.42±1.07 | 7.66±0.82 | 21.05±2.33 | 11.16±0.70 | 24.72±0.63 | 7.29±0.70 |
|  |  | 7.30 |  | 12.54 |  | 7.88 |
| b🡪c🡪e🡪g | 10.64±0.21 | 8.36±0.56 | 22.69±0.55 | 8.61±0.43 | 25.01±0.84 | 9.10±0.56 |
|  |  | **7.30** |  | **7.63** |  | **8.49** |
| a🡪c🡪e🡪g | 11.04±1.38 | 10.33±1.33 | 20.56±4.23 | 9.10±0.77 | 24.95±0.40 | 7.75±0.00 |
|  |  | **14.74** |  | **7.63** |  | **8.49** |

Bold type is the expected rip length calculated from the secondary structure predicted by Mfold software.

**5. Calculation of work and free energy corresponding to a single rip event**

Once RNA is opened, the tension first fall and then rises up again (Fig D). We selected fragments from where rip just begin (point A) to where the tension had just returned to its pre-drop level (point C). In order to reduce the effect of random noise, we calculate the area of the downward projection of the line ABC as the work W of the optical tweezers in this process. Because the tension between the starting point and the end point is equal, the state of the handle has not changed. The work of the tweezers can be divided into two parts: W_unfold_ provides the free energy and dissipation energy needed to open the RNA structure, and W_ss_ provides the free energy required for fully extending the single-stranded nucleotides released by the rip.

According to Bustamante’s work [2], the persistence length of single-stranded RNA is about 1 nm in the absence of magnesium ions. Therefore, we calculated W_ss_ directly with WLC model and deducted it from the work of optical tweezers. Then we subtracted W_ss_ from the word done by tweezers to calculate W_unfold_. Using the same method, we can get W_refold_ from RNA refolding curves.

A theory developed by Gavin Crooks [4] illustrated that, the following equation still stand even the experiment was performed far from quasistatic:

$e^{-\beta\Delta G}=\frac{\left\langle\left( 1+exp\left\{ \beta W+C \right\} \right)^{-1} \right\rangle_{U}}{\left\langle\left( 1+exp\left\{ \beta W-C \right\} \right)^{-1} \right\rangle_{F}}e^{C}$ （2）

While β=1/kBT, W is the work corresponding to RNA unfolding/refolding (positive for unfolding and negative for refolding); ΔG represents the free energy change during RNA unfolding; subscript U and F illustrate the unfolding and folding process; C=-βΔG+ln(nu/nf) where nu and nf are the amount of RNA unfolding and refolding curves respectively.

**6. Supplementary of small angle X-ray scattering**

SAXS experiments were performed at beamline BL19U2 of National Center for Protein Science Shanghai (NCPSS) at SSRF. The wavelength (λ) of X-ray radiation was set as 1.033 Å. Scattered X-ray intensities were collected using a Pilatus 1 M detector (DECTRIS Ltd). The sample-to-detector distance was set such that the detecting range of momentum transfer (q=4p sinq/l, where 2q is the scattering angle) of SAXS experiments was 0.01–0.45 Å−1. SAXS data were collected as 20 × 1 s exposures and scattering profiles for 20 passes were compared at 20 °C using 60 μl sample in 50 mM Tris-HCl, pH 7.0, 150 mM NaCl. Measurements were carried out at two different concentrations of 2 and 4 mg/ml. The data were analyzed in the ATSAS package [5] following the standard procedures. After subtracting buffer scattering, the data curves from different concentrations were scaled and merged using PRIMUS [6]. GNOM [7] was employed for estimating the particle maximum dimension (Dmax), real space Rg of the RNA, and calculation of the pair distance distribution function (PDDF). The ab initio envelopes of the folded rpoS RNAs were determined using DAMMIN [8] with 20 runs for each experimental group. DAMAVER was used to analyse the normalized spatial discrepancy between the 20 models. The filtered SAXS model by DAMFILT was showed in VMD. Linear Guinier plots in the Guinier region (q × Rg<1.3) were confirmed (Supplementary Section 4, Fig F- A).

RNA sample for SAXS was prepared by in-vitro transcription. The DNA template for transcription of rpoS 5′-UTR nucleotide 466-552 capped with two 5′-guanine and two 3′-cytosine was prepared by PCR amplification from the target plasmid using a forward (GAAATTAATACGACTCACTATAGGATCCGTAAACCCGCTGCGTTATTTCG) and a reverse (GGACCCGTGATCCCTTGACGGAACATTCAAGCAAA) primers. The in-vitro transcription mixture contained DNA template, 40 mM Tris, 10 mM DTT, 5 mM NTPs, 40 mM MgCl2, 1 mM spermidine, 0.01% (v/v) Triton X-100, 1 mg T7 RNA polymerase. The reaction was incubated in a water bath at 37 °C for 4 hours. The transcription product was precipitated with ethanol at -20 °C overnight, then dissolved in DEPC-treated water. The 91-nt RNA sample was separated by electrophoresis on urea-containing polyacrylamide denaturing gels and purified by Elutrap Electroelution System (GE Healthcare). Final RNA product was dialyzed into an SAXS buffer (10 mM NaH2PO4, 50 mM NaCl, pH 6.5) and quantified by absorbance at 260 nm.


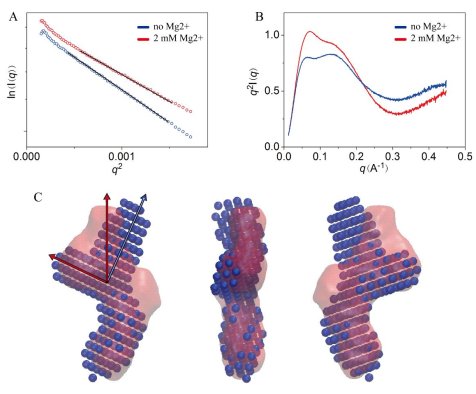


**Fig F. SAXS data analysis.** (A) Guinier region of the scattering curve with a linear fit line for *rpoS* inhibitory stem. (B) Kratky plot of *rpoS* inhibitory stem (C) The comparison of shape models of *rpoS* inhibitory stem in the absence (blue bead model) and presence (red envelope) of 2 mM Mg2+.

**Reference**

1. Nicholas MP, Rao L, Gennerich A. Covalent immobilization of microtubules on glass surfaces for molecular motor force measurements and other single-molecule assays. Mitosis: Springer; 2014. p. 137-69.

2. Liphardt J, Onoa B, Smith SB, Tinoco I, Bustamante C. Reversible unfolding of single RNA molecules by mechanical force. Science. 2001;292(5517):733-7. PubMed PMID: WOS:000168478300053.

3. Marko JF, Siggia ED. Stretching DNA. Macromolecules. 1995;28(26):8759-70. PubMed PMID: WOS:A1995TL65600008.

4. Crooks GE. Path-ensemble averages in systems driven far from equilibrium. Phys Rev E. 2000;61(3):2361-6. PubMed PMID: WOS:000085994000031.

5. Konarev PV, Petoukhov MV, Volkov VV, Svergun DI. ATSAS 2.1, a program package for small-angle scattering data analysis. J Appl Crystallogr. 2006;39:277-86. PubMed PMID: WOS:000235991200021.

6. Konarev PV, Volkov VV, Sokolova AV, Koch MHJ, Svergun DI. PRIMUS: a Windows PC-based system for small-angle scattering data analysis. J Appl Crystallogr. 2003;36:1277-82. PubMed PMID: WOS:000185178600026.

7. Semenyuk AV, Svergun DI. Gnom - a Program Package for Small-Angle Scattering Data-Processing. J Appl Crystallogr. 1991;24:537-40. PubMed PMID: WOS:A1991GL62900015.

8. Svergun DI. Restoring low resolution structure of biological macromolecules from solution scattering using simulated annealing. Biophys J. 1999;76(6):2879-86. doi: Doi 10.1016/S0006-3495(99)77443-6. PubMed PMID: WOS:000080556700002.
